# Does caffeine ingestion before a short-term sprint interval training promote body fat loss?

**DOI:** 10.1590/1414-431X20199169

**Published:** 2019-12-05

**Authors:** G.A. Ferreira, L.C. Felippe, R. Bertuzzi, D.J. Bishop, I.S. Ramos, F.R. De-Oliveira, A.E. Lima-Silva

**Affiliations:** 1 Grupo de Pesquisa em Ciências dos Esportes, Centro Acadêmico de Vitória, Universidade Federal de Pernambuco, Vitória de Santo Antão, PE, Brasil; 2 Grupo de Estudos em Desempenho Aeróbio da USP, Escola de Educação Física e Esporte, Universidade de São Paulo, São Paulo, SP, Brasil; 3 Institute for Health and Sport, Victoria University, Melbourne, Australia; 4 School of Medical and Health Sciences, Edith Cowan University, Perth, Australia; 5 Núcleo de Estudos do Movimento Humano, Departamento de Educação Física, Universidade Federal de Lavras, Lavras, MG, Brasil; 6 Grupo de Pesquisa em Performance Humana, Universidade Tecnológica Federal do Paraná, Curitiba, PR, Brasil

**Keywords:** High-intensity interval training, Body composition, Body fat distribution, Health, 1,3,7-Trimethylxanthine

## Abstract

We investigated the effect of caffeine ingestion combined with a 2-wk sprint interval training (SIT) on training-induced reductions in body adiposity. Twenty physically-active men ingested either 5 mg/kg of cellulose as a placebo (PLA, n=10) or 5 mg/kg of caffeine (CAF, n=10) 60 min before each SIT session (13×30 s sprint/15 s of rest). Body mass and skinfold thickness were measured pre- and post-training. Energy expenditure was measured at rest, during exercise, and 45 min after exercise in the first SIT session. Body fat was similar between PLA and CAF groups at pre-training (P>0.05). However, there was a significant decrease in body fat after training in the CAF group (−5.9±4.2%, P<0.05) but not in PLA (1.5±8.0%, P>0.05). There was no difference in energy expenditure at rest and during exercise between PLA and CAF groups (P>0.05), but the post-exercise energy expenditure was 18.3±21.4% greater in the CAF than in the PLA group (P<0.05). In conclusion, caffeine ingestion before SIT sessions induced a body fat loss that may be associated with higher post-exercise energy expenditure.

## Introduction

Sprint interval training (SIT) is defined as a model of exercise involving 5- to 30-s supramaximal efforts (>100% of maximal oxygen consumption; V̇O_2max_), interspaced by incomplete recovery periods (1). This type of training has been suggested as a time-efficient strategy to promote health benefits (1–3). It has been demonstrated that SIT increases V̇O_2max_ (2), ameliorates inflammation status (3), increases insulin sensitivity (2), and reduces systolic blood pressure (2). Furthermore, SIT has also been proposed to reduce body adiposity (4,5). It has been demonstrated that 4 weeks of SIT decrease the body fat of healthy young people by ∼4% (6). Part of the SIT-induced reduction in body fat is attributed to an increased post-exercise energy expenditure (4), which can contribute to a negative energy balance (7). In this sense, any additional agent able to stimulate post-exercise energy expenditure would be an attractive approach to speed up SIT-induced body fat loss.

A potent supplement able to increase post-exercise energy expenditure is caffeine (8–10). Previous findings have demonstrated that post-exercise energy expenditure after a 90-min moderate-intensity exercise session (55% of V̇O_2max_) was ∼27% higher when combined with caffeine ingestion one hour before the exercise (9). A caffeine-induced greater post-exercise energy expenditure (∼15%) also occurs when exercise intensity is increased to 75% V̇O_2max_ (8). It has been proposed that caffeine might increase post-exercise energy expenditure via a higher sympathetic nervous activation and/or a higher energy cost of ventilation (8,11). Combining caffeine ingestion and regular SIT might be an attractive approach to further speed up body fat loss and could have important practical implications for individuals who need to accelerate body fat loss (e.g., combat sports athletes and/or obese individuals). However, the effect of combining caffeine with short-term SIT is unknown.

Therefore, the aim of this study was to investigate the effect of caffeine ingestion before a single SIT session on energy expenditure (before, during, and post-exercise), and whether a 2-week SIT program combined with caffeine ingestion before each training session would lead to a larger reduction in body fat than a SIT program alone. We hypothesized that caffeine ingestion prior to SIT would stimulate greater post-exercise energy expenditure and subsequently increase training-induced body fat loss compared with SIT combined with a placebo.

## Material and Methods

### Participants

Twenty healthy, physically-active men (25.9±5.1 years; 176±9 cm; 75.6±10.0 kg; V̇O_2max_: 37.0±5.6 mL·kg^−1^·min^−1^) signed a consent form after being informed of the experimental procedures and possible risks involved in the study. The study was approved by the Ethics Committee for Human Research at the Federal University of Pernambuco as part of a larger thematic project, of which the remaining data are reported elsewhere (3).

### Experimental design

**Figure 1.**
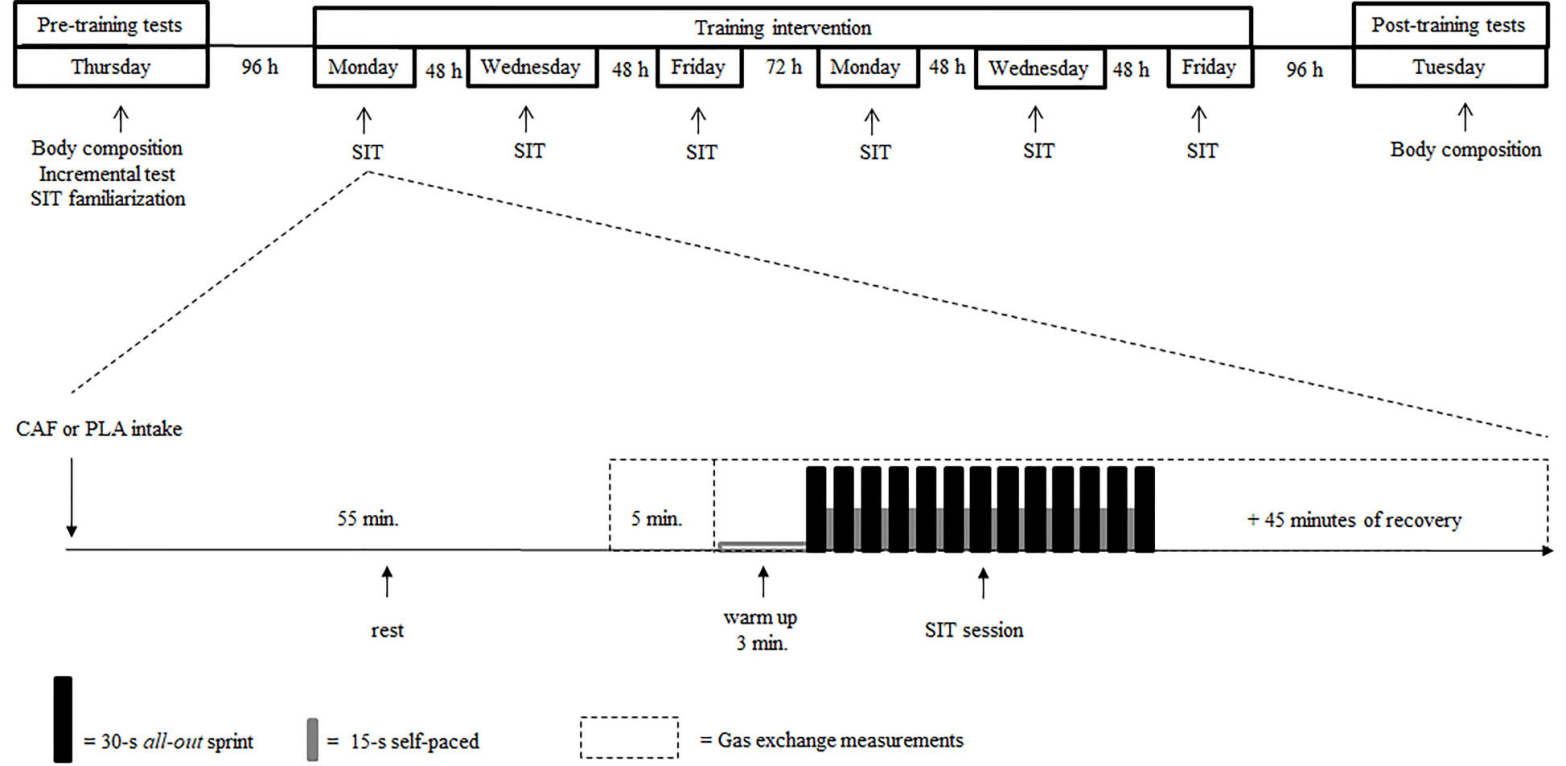
Experimental design of the study. SIT: Sprint interval training; CAF: caffeine; PLA: placebo.

In this placebo-controlled, double-blind study, the participants were randomly assigned using a block randomization technique to one of two groups: 1) CAF, who performed each SIT session after caffeine ingestion and 2) PLA, who performed each SIT session after placebo ingestion. Body composition assessment and an incremental test were performed 96 hours before the first SIT session (Figure 1). Familiarization with SIT was also performed 30 min after the incremental test. Participants performed six SIT sessions within two weeks and body composition was reassessed 96 h after the last training session.

A list of foods and beverages containing caffeine was given to each participant and they were instructed to refrain from foods or beverages containing caffeine during the 24 h prior to each test and training session. In addition, participants registered all foods and beverages ingested during the 24 h before the first visit and were asked to replicate their diet during the 24 h before subsequent tests and training sessions. Participants also filled a food diary during the 24 h before each training session to further check if they were maintaining the dietary recommendations. They received detailed verbal instructions about how to complete the food diary and were instructed to use household measurement devices to improve the precision of food quantification (12). They were asked to not perform exhaustive exercise or drink alcohol during the entire training period. They were also instructed to inform the investigators of any caffeine-related side effects during the training period. At the end of the training program, participants were asked to guess which supplement they thought they had ingested.

### Body composition

Height, body mass, and skinfold thickness from seven sites (chest, subscapular, midaxillary, triceps, abdominal, supra-iliac, and medial thigh) were assessed pre- and post-training. Skinfold thickness was measured three times in each site using a pre-calibrated skinfold caliper with jaw of ∼10 g/mm^2^, units of measurement of 0.1 mm and contact area of 90 mm^2^ (Sanny, AD 1007, Brazil). The median of three measures was calculated for each site and used for further analysis. Body density was calculated using a 7-skinfold equation (13), which was then converted to a body-fat percentage (14). All measurements were made by the same experienced evaluator who was blinded to which group participants were allocated. Skinfold thickness technique is a valid, practical, and inexpensive method to estimate body composition (15), and it is also used to determine the effect of training on body composition (5). The body mass index (BMI) was calculated by dividing body mass (kg) by height squared (m^2^). The sum of the seven skinfold thicknesses was also calculated.

### Incremental test

Participants performed an incremental test on a bicycle attached to a calibrated electromagnetically-braked roller (CompuTrainer Lab, RacerMate, USA) to determine their V̇O_2max_ and gas exchange threshold (GET). The test started with a 5-min warm-up at 70 W and then power was increased by 30 W every 3 min until exhaustion. A pedal cadence between 70–80 rpm was maintained throughout the trial; exhaustion was defined as an inability to maintain pedal cadence above 70 rpm. Gas exchange was measured breath-by-breath using an automatic metabolic cart, pre-calibrated according to manufacturer recommendations (Cortex Metalyzer 3B, Cortex Biophysik GmbH., Germany). Data were averaged every 30 s and the V̇O_2max_ was considered as the highest 30-s value during the test. The GET was determined by three investigators at the intensity corresponding to the lowest ventilatory equivalent of oxygen (V̇E/V̇O_2_) ratio (16).

### Supplementation

Participants in the caffeine group ingested a capsule containing 5 mg/kg of caffeine, while participants in the placebo group ingested a capsule containing 5 mg/kg of cellulose. The capsules containing caffeine or cellulose were ingested 1 h prior to each SIT session.

### Sprint interval training

SIT was performed on the same electromagnetically-braked roller used in the incremental test. After a 3-min warm-up at 90% of the GET, participants performed 13×30 s all-out sprints interspaced by 15 s of self-paced active recovery. Participants were encouraged to perform each sprint as fast as possible and to maintain a comfortable cadence during the recovery periods. The braking resistance was set up by inputting bicycle mass (9 kg) and the mass and height of the participant into the RacerMaterOne software (CompuTrainer Lab, RaceMate), which simulated a track with zero degrees of inclination. All training/trial sessions were performed between 17:00 to 18:00 h, at least 2 h after the last meal.

Gas exchange was monitored in the first training session at rest (5 min before SIT), during the entire exercise, and during the 45 min after the exercise session. The post-exercise energy expenditure was monitored throughout the 45 min post-exercise because this is the time in which the larger portion of excess post-exercise oxygen consumption occurs (17). Pre- and post-exercise measurements were made with the participants comfortably seated on a chair.

### Analyses

The V̇O_2_ and V̇CO_2_ were averaged during five minutes of rest (18), and the resting energy expenditure (REE) rate calculated using the following equation (19):

REE (kJ/min) = [(3.869×V̇O_2_ (L/min)) + (1.195×V̇CO_2_ (L/min))] × 4.184

The V̇O_2_ during the entire SIT (i.e., during the 13 sprints and active recoveries) and during the 45 min after exercise were averaged into 15-s intervals and the two areas under the V̇O_2_-time curve (exercise and post-exercise) were calculated using the trapezoidal method (18). Due to the limitations of using respiratory exchange ratio with a non-stable arterial CO_2_ partial pressure (17), as expected during and after SIT, the VO_2_ was converted to an energetic equivalent (kJ), assuming that 1 L of O_2_ is equal to 20.92 kJ (18). The total energy expenditure was defined as the sum of the exercise and post-exercise energy expenditure.

Food diaries were analyzed using a diet analysis software (Dietbox software, Mixpanel Mobile Analyst, USA).

### Statistical analysis

The Shapiro-Wilk test was used to check data distribution. Data from dietary intake were not normally distributed and were log transformed. Differences between PLA and CAF groups for pre-training characteristics of the participants and energy expenditure during the first training session were assessed using an independent *t*-test. Body composition changes from pre- to post-training were assessed using a two-way, mixed linear general model (group and time as factors). Dietary intake before SIT sessions was compared using a two-way, mixed linear general model (group and time as factors). Bonferroni *post hoc* test was used to locate any differences between groups and time points. Data are reported as means±SD. The level of significance was set at P≤0.05. All statistical procedures were performed in Statistic software version 10 (StataSoft Inc.^®^, USA).

## Results

### Baseline characteristics and dietary intake

There was no significant difference between PLA and CAF groups for any variable measured before training (all P>0.05, Table 1). There was no effect of group, training, or a group x training interaction for total energy intake or macronutrients distribution throughout the training period (all F_(1,50)_ <2.16, P>0.07, Table 2). Blinding was successful because 87% of the participants did not guess their supplement ingestion correctly. None of the participants reported any caffeine-related side effects.

**Table 1.** Pre-training characteristics of the participants.

	Placebo (n=10)	Caffeine (n=10)	P value for *t*-test
Age (years)	25.2±5.7	26.3±4.4	0.63
Height (m)	1.75±1.1	1.78±6.0	0.36
Body mass (kg)	73.2±10.8	78.1±8.4	0.28
Body mass index (kg/m^2^)	24.1±4.1	24.6±2.1	0.74
Body fat (%)	11.7±4.2	14.8±5.6	0.12
Body fat mass (kg)	8.7±4.1	11.8±5.1	0.12
Sum of seven skinfold thicknesses (mm)	88.8±30.7	114.5±48.1	0.10
V̇O_2max_ (L/min)	3.0±0.7	2.8±0.4	0.39
GET (L/min)	2.0±0.5	1.9±0.2	0.38

V̇O_2max_: maximal oxygen consumption; GET: gas exchange threshold. Data are reported as means±SD. There were no significant differences between the placebo and caffeine groups (P>0.05).

**Table 2.** Average of daily macronutrient and total energy intake during the training period for the placebo and caffeine groups.

	Placebo	Caffeine	P value for ANOVA
Mean carbohydrate (g/day)	276±43	238±96	0.99
Mean protein (g/day)	126±61	112±66	0.07
Mean lipid (g/day)	92±54	76±35	0.41
Mean total energy intake (kJ/day)	9645±3449	8702±3355	0.61

Data are reported as means±SD of the average of all six training sessions. ANOVA detected no effect of group, training, or group x training interaction for total energy intake or macronutrients distribution (P>0.05).

### Energy expenditure at the first SIT session

**Figure 2.**
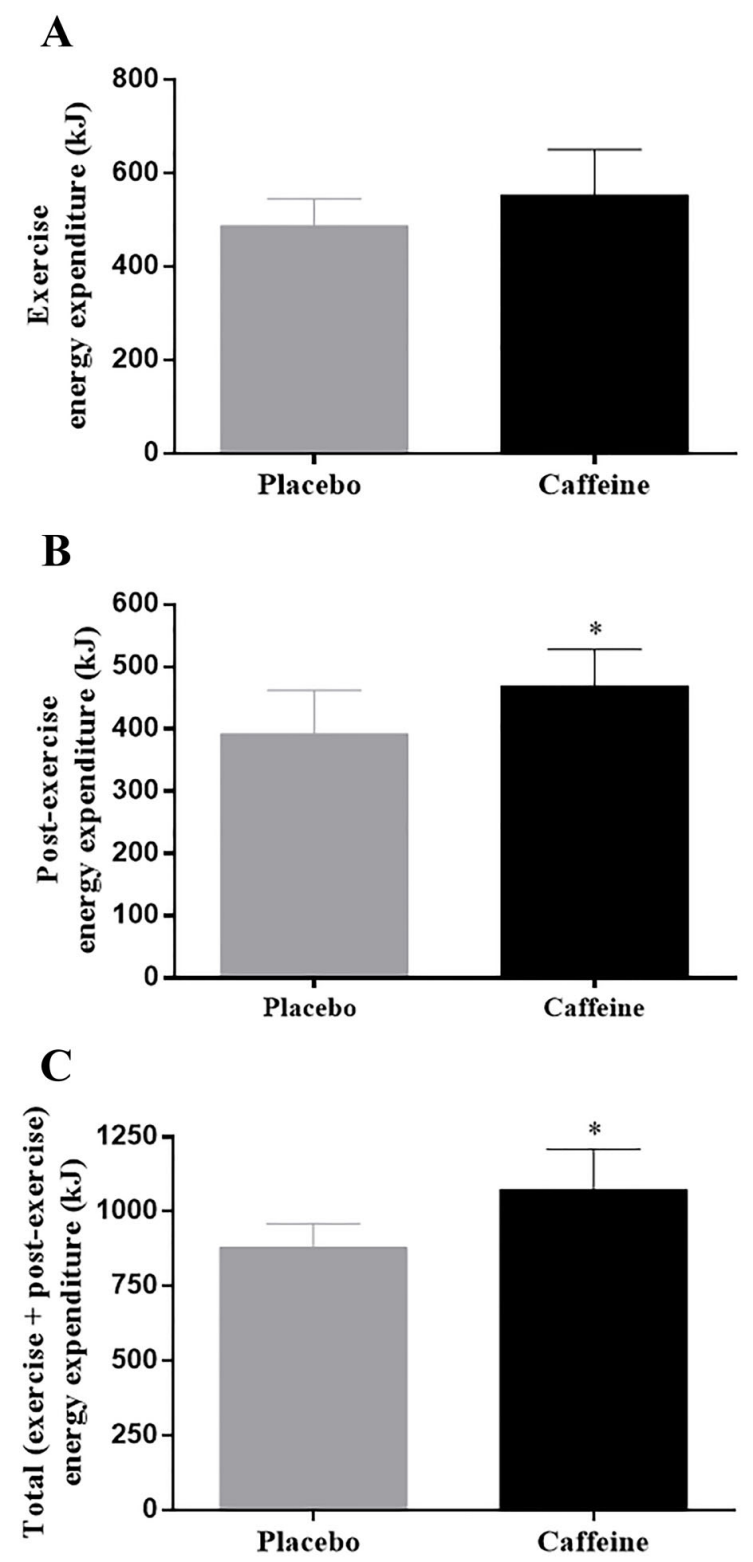
Energy expenditure during (**A**) and post (**B**) a single sprint interval training session, and total energy expenditure (exercise plus post-exercise; **C**) in the placebo and caffeine groups. *P<0.05 (independent *t*-test). Data are reported as means±SD, n=10 per group.

The resting energy expenditure was similar between the CAF and PLA groups (5.7±0.3 and 5.1±0.4 kJ/min, mean diff=0.6±0.5 kJ/min, 95%CI=−0.5 to 1.6, P=0.27). There was also no effect of caffeine ingestion on exercise energy expenditure, with similar values between CAF and PLA (552.7±98.4 and 487.5±56.2 kJ, mean diff=65.2±103.9 kJ, 95%CI=−10.5 to 140.8, P=0.09, Figure 2A). However, post-exercise energy expenditure was higher in the CAF than in the PLA group (469.2±58.6 and 392.0±70.3 kJ, mean diff=77.2±100.1 kJ, 95%CI=16.4 to 138.0, P=0.02, Figure 2B). Consequently, total energy expenditure (exercise + post-exercise) was higher in CAF than in PLA (1,073.3±135.5 and 879.6±80.6 kJ, mean diff=193.8±185.3 kJ, 95%CI=89.0 to 298.5, P=0.01, Figure 2C).

### Body composition changes with training

**Figure 3.**
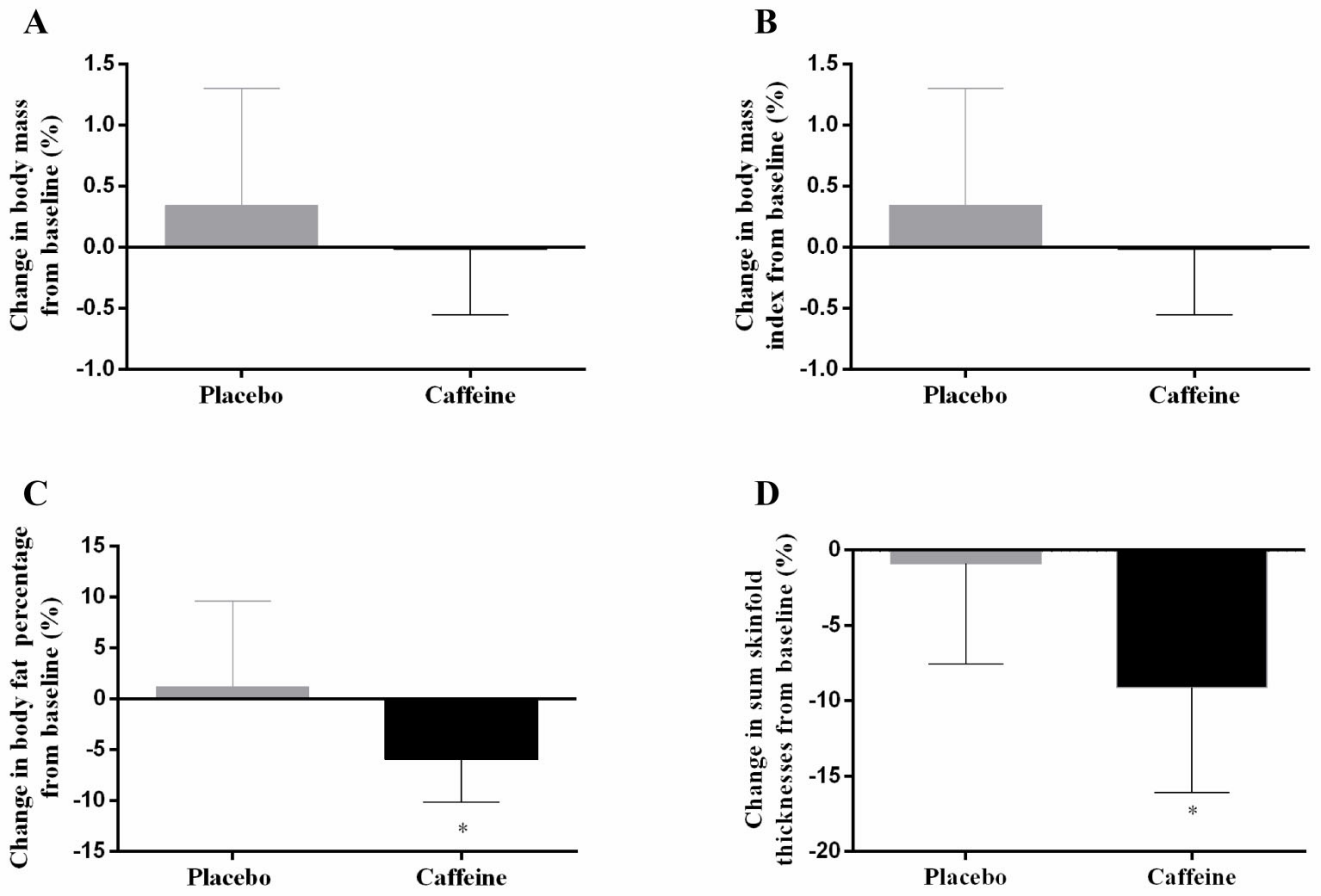
Percentage change from pre- (baseline) to post-training for body mass (**A**), body mass index (**B**), body fat percentage (**C**), and the sum of skinfold thicknesses (**D**) in the placebo and caffeine groups. *P<0.05 for group × time interaction, with values reducing from pre-training only in the caffeine group (two-way, mixed linear general model). Data are reported as means±SD, n=10 per group.

Body mass and BMI did not change with training in either the PLA or the CAF group (all F_(1,18)_ <0.53, P>0.47, Figure 3A and B). However, there was a significant group x time interaction for body fat percentage (F_(1,18)_=8.37, P=0.01, Figure 3C), in which values were reduced in CAF (mean diff=−1.0±0.7 %, 95%CI=−0.2 to −1.7, P=0.01, respectively) but not in PLA (mean diff=0.0±0.8 %, 95%CI=−0.7 to 0.8, p=0.99, respectively). Likewise, the sum of seven skinfold thicknesses decreased in CAF (mean diff=−9.1±7.0 mm, 95%CI=−2.7 to −15.5, P=0.01), but not in PLA (mean diff=0.9±6.6 mm, 95%CI=−5.5 to 7.3, P=0.99) (group x time interaction, F_(1,18)_=7.22, P=0.02, Figure 3D).

## Discussion

The main finding of the present study was that a 2-week SIT program induced a significant body fat loss when combined with caffeine, but not when combined with a placebo. These findings suggested that caffeine ingestion prior to exercise is useful to promote alterations in body composition within a short-term SIT program.

The lack of change in body fat combining SIT and placebo corroborates the results of a previous study showing that a 2-week SIT program did not promote any change in body fat (2). In contrast, reductions in body fat have been reported when the SIT intervention is longer (e.g., 4 weeks) (6). Thus, it appears that a SIT-induced reduction in body fat typically requires more than two weeks of training. However, in the present study, body fat was reduced by ∼6% after only two weeks of SIT when caffeine was ingested before each session. This finding suggests that caffeine ingestion before a regular SIT can accelerate SIT-induced reductions in body fat.

The mechanisms by which caffeine speeds up SIT-induced body fat reduction is not fully known and cannot be determined from our experimental design. However, caffeine significantly increased (∼20%) the post-exercise energy expenditure, which is accordance with previous studies showing that caffeine ingestion before moderate-intensity exercise increases post-exercise energy expenditure (8,9). The effect of caffeine on post-exercise energy expenditure may have been larger than what we observed as caffeine half-life is approximately 5 to 6 h (20); therefore, caffeine may have acted to increase energy expenditure beyond 45 min of recovery. In support of this, energy expenditure did not return to basal levels within 45 min of recovery. Assuming that the increase in post-exercise energy expenditure with caffeine remained throughout the six SIT sessions, this may have promoted a negative energy balance leading to a greater body fat loss. However, further studies monitoring post-exercise energy expenditure for longer times (e.g., over 24 h) and after all SIT sessions will be necessary to confirm this hypothesis.

While post-exercise energy expenditure was increased, the exercise energy expenditure was not significantly altered with caffeine ingestion. It has been demonstrated that caffeine ingestion before 60 min of moderate exercise (∼65% of maximal aerobic power) and/or a bout of high-intensity exercise (∼90% V̇O_2max_) increases energy expenditure (11,21). However, caffeine ingestion failed to significantly increase energy expenditure during repeated Wingate bouts (22). This suggests that caffeine ingestion does not significantly increase exercise energy expenditure during a session of SIT, perhaps because in this model of exercise individuals reach their V̇O_2max_ rapidly within one or two sprints, and this limits any further increase in energy expenditure (3,22). Therefore, the combined effect of caffeine and SIT on changes in body composition does not seem to be associated with greater exercise energy expenditure.

Another interesting effect of caffeine is a suppression of appetite (11). As participants were asked to maintain their habitual food intake, energy intake did not change statistically throughout the training period in both groups. However, it is noteworthy that energy intake seems to be slightly lower in the CAF than in the PLA group (∼8%). This small, non-significant reduction in daily energy intake when summed over the 2-week intervention might have impacted on body fat reduction. Further studies should explore the role of caffeine-induced reduction in appetite on body fat reduction using a “free” food intake design.

The present study had some limitations. Although skinfold thickness is a valid and widely used method to estimate body composition (5,15), more accurate techniques such as DEXA would have been suitable (23). However, the same experienced investigator, who was blinded to which supplement participants ingested, performed all measurements; therefore, any error of measurement should have been similar across the groups. Nevertheless, further studies using DEXA may provide more accurate estimates of changes in body composition. In addition, we recruited healthy men with normal BMI; therefore, these findings cannot be extended to other populations (e.g., obese individuals). New studies in obese individuals would be helpful to understand the potential of caffeine in the treatment of obesity and associated diseases. Finally, although participants were randomly allocated into the groups and baseline body mass and body fat were not statistically different between the groups, values were somewhat higher in the CAF group, which may have had an impact on our results.

The findings of the present study provide the first evidence that caffeine ingestion before SIT sessions might be useful to promote a reduction in adiposity in a shorter time period and promote health benefits associated with a reduction in adiposity. As caffeine intake combined with SIT might have the potential to augment training-induced decreases in body fat, it would be helpful for individuals who need to accelerate body fat loss. In addition, new studies assessing the effect of caffeine intake combined with SIT on body composition in obese individuals would help the understanding of the potential of caffeine in the treatment of diseases associated with obesity.
